# Setting the standard: empirical benchmarks for case-level site–central discordance in radiographic endpoints in oncology clinical trials

**DOI:** 10.1016/j.conctc.2026.101682

**Published:** 2026-09-03

**Authors:** Gregory V. Goldmacher, Cynthia Muller Goldberg, Leslie Boyle, Liyi Jia, Christine K. Gause, Marjorie C. Green, Lawrence H. Schwartz

**Affiliations:** a Clinical Imaging & Pathology, Merck & Co., Inc., Rahway, NJ, USA; b Biostatistics and Research Decision Sciences, Merck & Co., Inc., Rahway, NJ, USA; c Global Clinical Development, Merck & Co., Inc., Rahway, NJ, USA; d Department of Radiology, Memorial Sloan Kettering Cancer Center, New York, NY, USA

**Keywords:** Blinded independent central review, Discordance, Investigator review, Oncology, Radiology, RECIST

## Abstract

**Background:**

Progression-free survival and objective response rate assessed per Response Evaluation Criteria in Solid Tumors, version 1.1 (RECIST v1.1), are based on serial radiographic assessments of tumor burden. We quantified case-level discordance between investigator and blinded independent central review (BICR)-determined progressive disease (PD) and objective response (OR) to provide benchmarks that can help clinical study teams judge whether observed discordance rates are expected or a data quality concern.

**Methods:**

We performed a pooled analysis of 39 phase 2 and 3 solid-tumor trials of pembrolizumab-based regimens in which radiographic images were reviewed by investigators and BICR and RECIST v1.1 was used to assess response. Case-level discordance of PD, confirmed OR, and unconfirmed OR were calculated from 2×2 tables.

**Results:**

Case-level discordance was 17.7% (95% CI 17.2–18.2) for PD (n = 20,908), 12.2% (11.8–12.7) for confirmed OR (n = 21,520), and 14.7% (14.1–15.3) for unconfirmed OR (n = 15,209). Discordance varied by cancer type, ranging from 11.8% (melanoma) to 22.8% (hepatocellular carcinoma [HCC]) for PD, from 5.4% (HCC) to 20.0% (cervical cancer) for confirmed OR, and from 6.3% (HCC) to 19.7% (cervical cancer) for unconfirmed OR. Trials with real-time verification of progression showed lower PD discordance than those without (17.3% [95% CI 16.7-17.8] vs 20.2% [18.8-21.7]). PD discordance was similar in double-blind and open-label trials (17.6% [95% CI 16.9-18.3] vs 17.8% [17.0-18.6]). Confirmed OR discordance was more common in double-blind trials (13.3% [12.7-13.9] vs 10.9% [10.3-11.6]).

**Conclusion:**

This analysis of trials of pembrolizumab-based regimens addresses a critical gap in oncology trial methodology by establishing empirical benchmarks for site–central discordance of RECIST v1.1 response assessment, enabling study teams to distinguish expected variability from potential data quality signals. While the underlying sources of discordance characterized here are not specific to immunotherapy, the reported magnitude of discordance should be applied cautiously to trials of non-immunotherapy agents.

## Highlights

• Site and central radiography reviewers often differ in assessing tumor response. [82 characters].
• Progressive disease and objective response discordance varies across cancer types. [84 characters].
• Access to clinical context likely influences how progression is interpreted. [78 characters].
• Our analysis provides oncology clinical trialists with discordance rate benchmarks. [85 characters with spaces].

## 1 Introduction

Progression-free survival (PFS), objective response rate (ORR), and duration of response are clinically meaningful endpoints that inform pipeline-related decisions and can lead to or support regulatory approval of oncology drugs. In solid tumor indications, these endpoints are typically based on serial radiographic assessments of changes in tumor burden over time guided by rules called response criteria. The most common, well established response criteria are the Response Evaluation Criteria In Solid Tumors, version 1.1 (RECIST v1.1) [1]. Serial radiographic assessments may be done locally by site investigators or by blinded independent central reviewers (BICR) in academic or commercial imaging core laboratories. In many clinical trials, especially those with registration intent, assessments are performed both locally and centrally.

Investigators and independent reviewers sometimes disagree on the determination of response and progression in clinical trial participants [[2], [3], [4], [5]]. A high degree of discordance between site and central reviewers can be a sign of reviewer bias, especially in an open-label study, or of low data quality, and it is thus scrutinized by clinical trial sponsors, and occasionally by regulatory authorities, as an indirect indicator of data integrity risk. However, factors other than bias or low quality can contribute to discordance, including the tumor type, imaging method, reviewer training, and magnitude of response to treatment.

Discordance between radiologists is a well-studied phenomenon. The factors that impact the rates at which expert radiologists disagree with each other in general, and in oncology endpoint assessments in particular, have been the subject of extensive study [6,7]. These factors include those related to technical aspects of image review, human judgment, and the radiographic manifestations of particular cancer types, as well as the overall magnitude of response. Because BICR assessment is often done using multiple reviewers working independently of one other, there is ample opportunity to evaluate discordance in that setting, and reader variability has been evaluated systematically across many trials to quantify rates of disagreement between central reviewers [8]. However, discordance between site and central reviewers may be greater because of investigator access to clinical information that can influence their assessment of radiographic findings, differences in level of training on response criteria, and differences in the read systems used to perform the assessment. Site-central discordance has been evaluated across multiple trials at the level of treatment groups and intergroup comparisons. These analyses have generally shown that even though PFS assessments between site and central reviewers may be discordant, there is a strong concordance in the hazard ratios for PFS based on site and central reviews in randomized trials [[9], [10], [11], [12], [13], [14]]. Sites tend to overestimate ORR relative to BICR in phase 2 trials, which are typically non-randomized and open label [3,4]. However, there has not been a systematic assessment of site-central discordance across tumor types at the individual participant level. As a result, trial management teams review discordance in ongoing trials without an evidence-based understanding of what levels of discordance should be expected.

The lack of empirical benchmarks for discordance has practical consequences for clinical trial management teams that routinely monitor site–central discordance as part of ongoing data quality oversight. When discordance rates appear elevated, teams may invest significant time and resources investigating root causes, retraining investigators, or questioning the integrity of the central review process; this is often done without a clear basis for determining whether the observed rate is actually unusual. Conversely, genuinely anomalous discordance may be unrecognized if there is no frame of reference for what is typical. Tumor-specific, case-level benchmarks would enable a more evidence-based approach to discordance monitoring, helping clinical trial teams focus their attention where it is most warranted. We analyzed case-level discordance of radiographic response and progression between site and central reviewers in a large oncology clinical trial database that includes multiple tumor types to establish empirical benchmarks that can inform evidence-based discordance monitoring in clinical trials.

## 2 Methods

### 2.1 Studies

This pooled analysis included select phase 1b, 2, and 3 clinical trials of participants with solid tumors that were initiated by Merck Sharp & Dohme LLC, a subsidiary of Merck & Co., Inc., Rahway, NJ, USA (“sponsor”), between 2012 and 2020. Clinical trials were included regardless of therapeutic mechanism of action or trial outcome as long as radiographic images were reviewed by both investigators and independent central reviewers and all reviewers used RECIST v1.1 to determine treatment response. The protocols and amendments of each included study were approved by the appropriate ethics body for each participating center. Participants in all clinical trials provided written informed consent.

### 2.2 Radiographic review

Response to treatment in all included trials was assessed according to RECIST v1.1 with a single modification that allowed investigators and central reviewers to select up to 10 target lesions and up to 5 lesions per organ instead of 5 total lesions and 2 per organ if they wish to include a broader sample of the total tumor burden; all other elements of RECIST v1.1 were implemented without modification, including short-axis measurement and 15 mm/10 mm handling of lymph nodes, the 20% plus 5-mm progression rule, and the definitions of new lesions and non-target progression [1]. For trials of prostate cancer, response in soft-tissue lesions was assessed using RECIST v1.1 and response in bone lesions observed on bone scans was assessed using Prostate Cancer Working Group 3 criteria [15]. irRECIST [16] was used by investigators in some trials, and immune-related response criteria (irRC) [17] were used by central reviewers in other select trials; iRECIST [18] was not used in any study. For each timepoint, reviewers determined an overall response of complete response (CR), partial response (PR), stable disease (SD), progressive disease (PD), or nonevaluable. If participants had only non-measurable lesions before study treatment initiation, the timepoint response of “non-CR/non-PD” was considered equivalent to SD.

Central radiographic review was performed by commercial imaging core laboratories. The reads followed a “2 + 1” paradigm, with two primary readers and adjudication by a third reader if the primary readers disagreed on an adjudication trigger. Adjudication triggers were defined in the independent imaging review charter for each study but typically included disagreement on the date of progression, the best overall response (with confirmation of PR and CR), and the initial date of objective response (OR). Central reviewers were drawn from a small pool of radiologists (typically 4 to 10) selected for each trial. Any member of the pool could act as a primary reader or as the adjudicator on any given case. All reviewers were US board-certified radiologists (or European equivalent) with experience in reading clinical trial images and had documented experience with the specific tumor type investigated in the trial. Each reviewer of a trial was trained on the response criteria and relevant protocol specifics. The need for retraining during a trial was based on the results of a reader monitoring process agreed upon by the sponsor and core lab scientific teams. The reader monitoring process included intra-reader variability assessment (i.e., reintroducing previously read cases to a reader after a washout period) and assessment of adjudication rates and adjudicator selection rates (e.g., a root cause analysis based on sufficient data could identify the need for retraining if a significant increase in adjudication rates was observed). Retraining included a review of the previously presented read rules and a qualitative discussion of select locked cases that required adjudication.

Local review was performed by investigators in collaboration with their local radiologists. Different assessors could conduct the reads at different timepoints. The sponsor provided read criteria training during investigator meetings or through an online learning management system. Investigators determined overall response at each timepoint. Outcomes that contributed to the determination of clinical trial endpoints (e.g., date of progression and best overall response), were calculated by sponsor statisticians based on predefined rules. Most phase 3 clinical trials included real-time verification of progression (VOP) review whereby rapid BICR was performed when an investigator identified radiographic progression. The investigator was informed of whether progression was verified by the central reviewer and if not, the investigator was asked to consider continuing treatment and imaging until PD was verified centrally; the investigator was not asked to change their response assessment to match the central assessment.

Read platforms were not standardized. Each of the central imaging core laboratories used their own picture archiving and communication system (PACS) integrated with an electronic data capture system; some of these provided semi-automated measurement support (e.g., extraction of longest and perpendicular diameters from a reader-drawn lesion outline) and computational support for RECIST calculations (e.g., sums of diameters and their change over time), while others did not. Investigators recorded assessments using the clinical PACS and local instruments in use at each site; measurement automation was varied.

### 2.3 Determination of discordance

All investigator and central reviewer assessments used in this analysis were performed using RECIST v1.1; reads based on irRECIST and irRC were excluded because they were exploratory, applied in only a subset of studies, and the same modified criteria were never used by both site and central reviewers in any single trial. For each participant, the occurrence or non-occurrence of progression and OR was determined from both investigator and central image reviews. For progression, the determination was whether an overall response of PD was observed at any visit. OR was defined as a best overall response of PR or CR. In most trials, objective response determined by central review required confirmation of PR or CR on a scan acquired at least 4 weeks after the initial scan that showed response. The same rule was applied when sponsor statisticians derived the best OR from the sequence of visit responses provided by the investigator. An additional analysis of OR without confirmation was performed on a subset of trials. In this analysis, OR for both investigator and central review was determined by the sponsor statistical team by selecting the best overall response from the sequence of timepoint responses without requiring confirmation of PR or CR.

**Table:** Case-level discordance of PD and OR were calculated using a 2 x 2 table for each tumor type.

		Investigator-assessed PD/OR		Total
		Yes	No	
**BICR-assessed PD/OR**	**Yes**	a	b	n1.
	**No**	c	d	n2.
**Total**		n.1	n.2	n

Discordance rate (%) = (b + c)/(a+b + c + d)*100.

The analysis included all participants with that tumor type pooled across all trials conducted in that tumor type, regardless of treatment arm or line of therapy. Only participants who had at least one evaluable post-baseline imaging assessment for which both investigator and BICR results were available were included in the analysis. The rate of discordance was defined as the number of cases for whom the investigator and central review disagreed on the outcome of interest divided by the total number of cases; 95% confidence intervals (CIs) were assessed using the Clopper–Pearson method. Case-level discordance of PD and OR were also calculated for the aggregate of all tumor types, the aggregate of all open-label trials, and the aggregate of all double-blind trials. Case-level discordance of PD was additionally calculated for the aggregate of all trials that used real-time VOP and for the aggregate of all trials that did not use VOP. The directionality of PD and confirmed OR differences was assessed descriptively by splitting each 2 x 2 table into its two off-diagonal cells of investigator-positive/BICR-negative and BICR-positive/investigator-negative and calculating the percentage of discordant cases that were investigator-positive/BICR-negative and the percentage that were BICR-positive/investigator-negative. Analyses by trial or treatment arm were not performed because the focus of the analysis was reviewer variability.

## 3 Results

A total of 39 clinical trials were included in the analysis (Table A.1). The 12 cancer types represented in the analysis were cervical, colorectal, esophageal, gastric, head and neck (squamous), hepatocellular, lung, melanoma, prostate, renal cell, triple-negative breast, and urothelial. Table 1 summarizes the number of cases and trials by cancer type that were used to assess discordance of PD and of OR.

**Table 1:** Summary of cases and trials included in the analyses of progressive disease (PD) and objective response (OR) discordance by cancer type.

Cancer Type	Assessed for Discordance of PD		Assessed for Discordance of OR (With Confirmation)		Assessed for Discordance of OR (Without Confirmation)	
	No. Trials	No. Cases	No. Trials	No. Cases	No. Trials	No. Cases
Cervical	1	565	1	579	1	579
Colorectal	2	369	2	403	2	403
Esophageal	3	1319	3	1328	3	1330
Gastric	5	3554	5	3795	2	2280
Head and neck (squamous)	2	1171	2	1168	2	1171
Hepatocellular	2	545	2	496	2	496
Lung	8	3541	8	3911	5	3329
Melanoma	4	1343	3	1262	2	1258
Prostate	4	4210	4	4216	0	0
Renal cell	2	967	2	1075	2	1075
Triple-negative breast	3	1644	3	1576	3	1576
Urothelial	3	1680	3	1711	3	1712

**Fig. 1:**
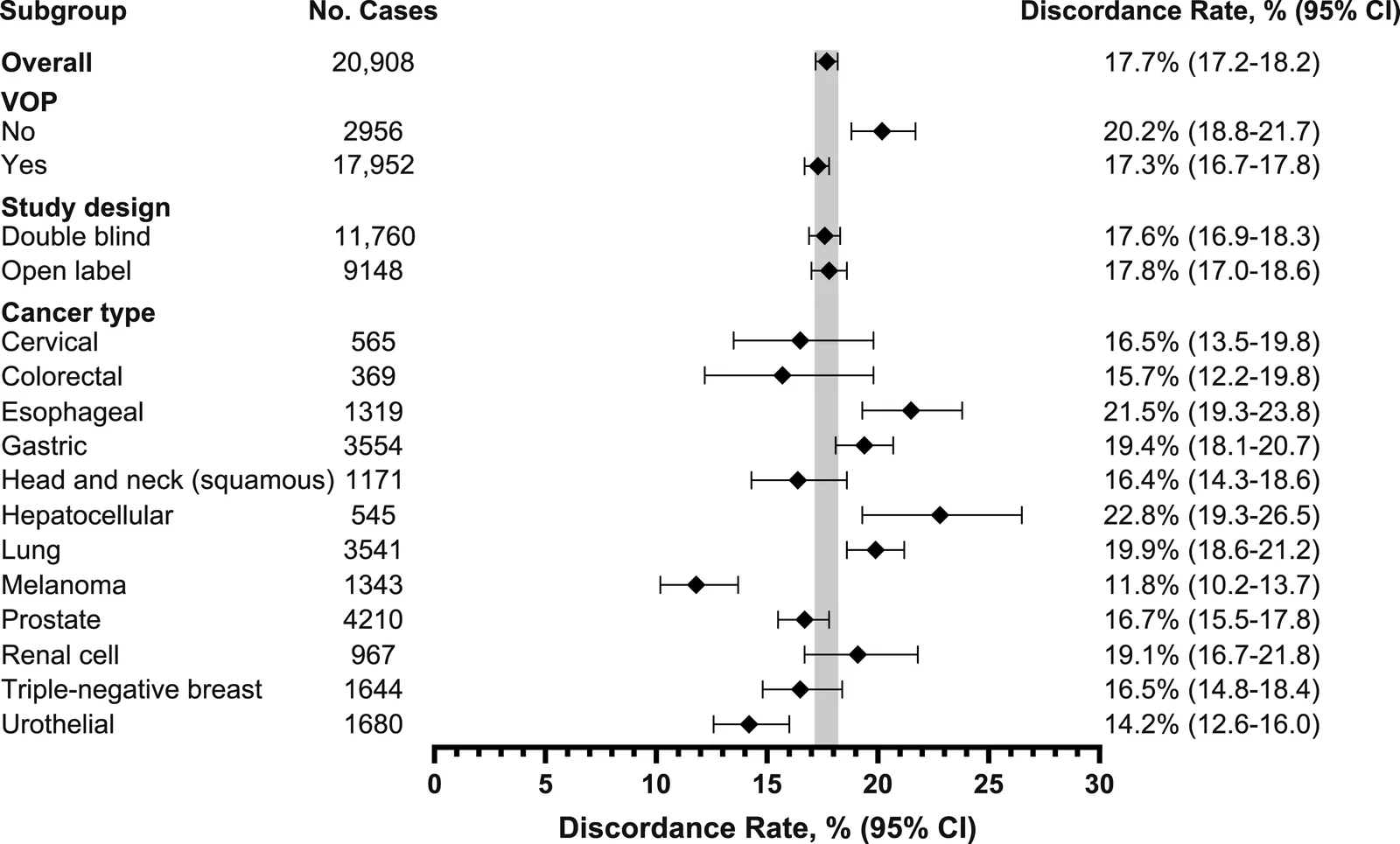

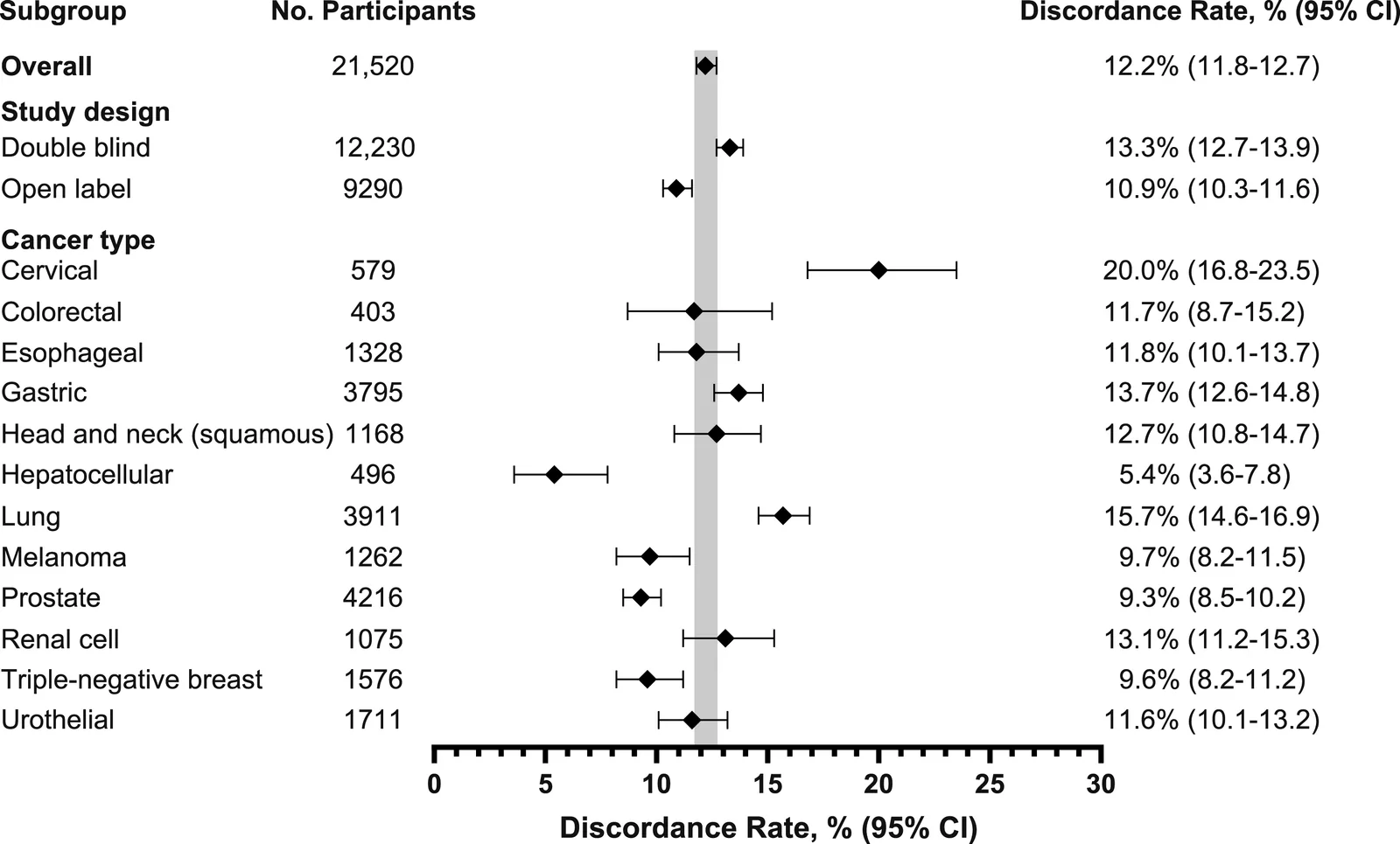

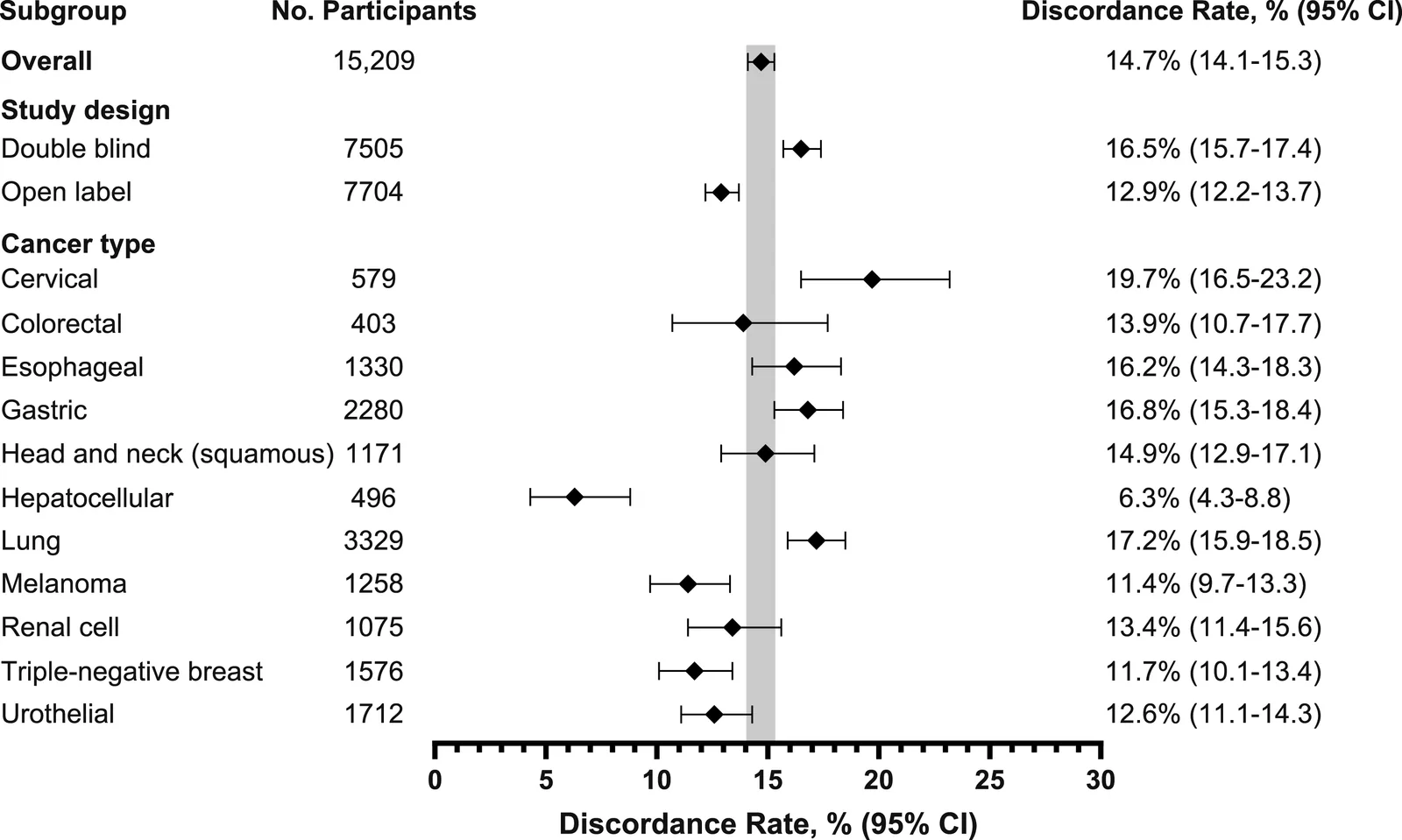
Summary of case-level rates of discordance between investigators and blinded, independent central radiologists. Abbreviations: VOP, verification of progression. **A.** Discordance of progressive disease. **B.** Discordance of objective response with confirmation. **C.** Discordance of objective response without confirmation.

Discordance of PD between investigator and central review across trials was 17.7% (95% CI 17.2-18.2) among 20,908 cases, ranging from 11.8% (10.2-13.7) in melanoma to 22.8% (19.3-26.5) in hepatocellular carcinoma (Fig. 1A, Table A.2). There was no difference in PD discordance between double-blind and open-label trials (17.6% [95% CI 16.9-18.3] and 17.8% [17.0-18.6], respectively). Trials with real-time VOP showed lower PD discordance than trials without real-time VOP (17.3% [95% CI 16.7-17.8] and 20.2 [18.8-21.7], respectively).

The confirmed ORR per BICR by tumor type ranged from 15.1% for hepatocellular carcinoma to 61.5% for cervical carcinoma (Table 2). Discordance of OR with confirmation across trials was 12.2% (95% CI 11.8-12.7) among 21,520 cases and was highest in cervical cancer (20.0% [95% CI 16.8-23.5]) and lowest in hepatocellular carcinoma (5.4% [3.6-7.8]) (Fig. 1B–Table A.3). There was a higher discordance of OR with confirmation in double-blind trials (13.3% [95% CI 12.7-13.9]) compared with open-label trials (10.9% [10.3-11.6]).

**Table 2:** Summary of confirmed objective response rate (ORR) per blinded, independent central review by cancer type.

Cancer Type	No. Participants	No. Responders	Confirmed ORR
Cervical	579	356	61.5%
Colorectal	403	162	40.2%
Esophageal	1328	345	26.0%
Gastric	3795	1591	41.9%
Head and neck (squamous)	1168	303	25.9%
Hepatocellular	496	75	15.1%
Lung	3911	1838	47.0%
Melanoma	1262	351	27.8%
Prostate	4216	674	16.0%
Renal cell	1075	515	47.9%
Triple-negative breast	1576	423	26.8%
Urothelial	1711	636	37.2%

Discordance of OR without confirmation across trials was 14.7% (95% CI 14.1-15.3) among 15,209 cases (Fig. 1C–Table A.4). Similar to the analysis of discordance of OR with confirmation, discordance of OR without confirmation was highest in cervical cancer (19.7% [95% CI 16.5-23.2]), lowest in hepatocellular carcinoma (6.3% [4.3-8.8]), and higher in double-blind versus open-label trials (16.5% [15.7-17.4] vs 12.9% [12.2-13.7]).

The direction of discordance was informative (Table A.5). For PD, investigators recorded PD not confirmed by central review roughly twice as often as the reverse (66% of PD-discordant cases overall). The only cancers for which central review recorded PD more often than investigators were melanoma and prostate cancer. Discordance of confirmed OR showed no consistent directional bias, with investigator-excess and central-excess cases approximately equal overall.

## 4 Discussion

In this review of case-level data from aggregated clinical trials of pembrolizumab given as monotherapy or in combination with cytotoxic or targeted agents in participants with solid tumors, we observed that site and central reviews based on RECIST v1.1 were discordant for PD in 17.7% of cases, discordant for OR with confirmation in 12.2% of cases, and discordant for OR without confirmation in 14.7% of cases. Documented factors that increase the likelihood of discordance include heterogeneity of response among lesions, the total number of lesions (more lesions increase the probability of site and central reviewers choosing different lesions that respond differently), how close the “true” average changes in tumor size are to the thresholds specified in RECIST v1.1, and the qualitative judgement inherent in assessment of non-target and new-lesion assessment [2,7]. These factors can produce genuine disagreement between two competent readers without either being in error.

Discordance of both PD and OR varied substantially across cancer types. This likely reflects, in part, differences in the measurement reproducibility of the lesion types characteristic of each tumor, rather than differences in radiologist expertise. Peritoneal and nodal disease, common in cancers such as ovarian and gastric cancer, are intrinsically harder to measure reproducibly, even for experienced readers, than lesions in solid organs such as liver and lung. Peritoneal implants often have irregular, poorly defined margins affected by bowel distension and peristalsis, which vary between imaging timepoints and between readers; nodal measurement depends on precise localization of the short axis, which is more sensitive to slice selection than measurement of a discrete mass. Prior studies have documented substantially lower interobserver measurement concordance for lymph node lesions and lower lesion-selection reproducibility for peritoneal lesions than for other lesion types [19], consistent with a broader literature showing that interobserver variability in RECIST-based measurements can be large enough to affect response classification [20].

The magnitude of cross-tumor variation observed in this analysis, including a nearly four-fold difference in confirmed OR discordance between hepatocellular carcinoma (5.4%) and cervical cancer (20.0%), provides evidence that a single, tumor-agnostic threshold for “acceptable” discordance in response assessment based on RECIST v1.1 is inappropriate. Instead, our data suggest that expectations should be calibrated to the specific tumor type, as the biological and radiographic characteristics of each cancer create different baseline levels of interpretive complexity when applying RECIST v1.1. The low OR discordance observed for hepatocellular carcinoma likely reflects, at least in part, the ORR observed in this tumor type, which was the lowest of any tumor type in our dataset, rather than technical ease of assessment. Response rate and response discordance were closely correlated across the 12 tumors in our analysis, suggesting that when there are few patients who experience response, there are correspondingly few borderline cases in which investigator and central measurements fall close enough to a threshold to disagree. A further contributing factor specific to hepatocellular carcinoma is that RECIST v1.1 scores response based on change in overall lesion diameter without regard for the vascular changes that treatment commonly produces in this tumor. Hepatocellular carcinoma lesions frequently show reduced arterial enhancement (devascularization or necrosis) as an early treatment effect, often with only modest or delayed change in overall size; because RECIST v1.1 does not capture this, size-based measurement may under-detect treatment effect relative to a criterion that credits loss of viable, enhancing tumor. This is the rationale underlying modified RECIST (mRECIST) [21], which restricts measurements to the enhancing portion of the lesion. We do not suggest that vascularity in hepatocellular carcinoma is difficult to visualize; standard multiphase CT and MR protocols readily demonstrate arterial hyperenhancement and washout. Rather, the arterial phase in which viable HCC is most conspicuous is a brief, hemodynamically sensitive window, the optimal timing of which depends on patient circulation, contrast bolus characteristics, and injection protocol. These factors may introduce an additional, hepatocellular carcinoma-specific source of measurement variability distinct from any difficulty in assessing vascularity itself.

A fundamental difference between site and central interpretation of response is that investigators have access to participants’ clinical data in addition to their imaging results. This may explain why discordance was more likely for PD than for OR and why investigators were more likely to call PD than central reviewers for most tumor types. For example, an investigator may assess a radiographically ambiguous new liver lesion as “unequivocal” in the presence of newly elevated liver enzymes. Although the availability of clinical information is more likely to result in an investigator but not a central reviewer calling PD, there are times when the reverse may be true. For example, a central reviewer may call a small or indeterminate new pulmonary lesion as progression when the site reviewer, aware of concurrent infection or other benign explanation would not. The lack of consistent directional bias in confirmed OR disagreement suggests that as would be expected, the clinical-information effect impacts calls of progression but not response. BICR can be designed to include prespecified clinical information that is incorporated into the review by an oncologist after the radiology read is complete. The melanoma trials were the only studies in our dataset that incorporated clinical information (in the form of cutaneous lesions observed on physical examination) in the BICR reads. It is interesting to note that the PD discordance rate among cancer types was lowest for melanoma (11.8%) in our analysis and that melanoma was only one of two cancers for which central review called PD more often than investigators. Other factors that may increase the potential for discordance of PD is that progression may be based on target lesion measurements, the identification of new lesions, or qualitative assessment of progression. The other tumor type for which PD was called more frequently by central reviewers than investigators was prostate cancer, likely because of the prevalence of bone metastases and the RECIST v1.1–PCWG3 pairing.

One mechanism that clinical trial sponsors use to minimize the potential for overcalling PD by investigators is to institute processes for real-time VOP by BICR. Because real-time reads increase the cost and operational complexity of clinical trials, sponsors may question whether they are worth the effort. Our results show that real-time VOP by BICR reduces discordance by approximately 3 percentage points (from 20.2% to 17.3%). However, this may not capture the full value of real-time VOP because cases where VOP prevents discordance may have a disproportionately large impact on study data by making it more likely that progression that is radiographically less obvious is identified by BICR.

The practical significance of these findings lies in their potential to transform how discordance is interpreted during trial conduct by providing benchmarks for trial teams. Currently, when a trial team observes a given discordance rate, for example, a 22% PD discordance rate, there is no systematic way to determine whether this is cause for concern or a reflection of the expected variability for that tumor type. Our results suggest that a 22% PD discordance rate would be unremarkable in hepatocellular carcinoma (observed discordance rate of 22.8%) but would be notably elevated in melanoma (observed discordance rate of 11.8%). Without benchmarks, the same discordance rate could thus trigger unnecessary remedial action in one context but fail to prompt warranted investigation in another. It is important to note that discordance is not inherently a marker of poor study conduct or specific to any tumor type. Well-understood factors such as the aforementioned differences in access to clinical information, variability in lesion selection, the subjectivity of non-target disease assessment, and the proximity of true tumor changes to RECIST thresholds all contribute to discordance. However, discordance that substantially exceeds the benchmarks reported here, particularly after accounting for tumor type and trial design, may signal issues such as inadequate investigator training, protocol noncompliance, or systematic differences in image interpretation that merit further evaluation. In this way, benchmarks serve both to reassure teams when discordance is within expected bounds and to sharpen their focus when it is not.

There was no difference in PD discordance between double-blind and open-label clinical trials. Discordance of OR, both with and without confirmation, was higher in double-blind trials than in open-label trials. There is no clear explanation for this finding. Ultimately, many factors remain uncontrolled in such a subgroup analysis, making it difficult to determine whether the finding of higher OR discordance in double-blind trials is a true difference or merely due to chance. To establish confidence in this finding, replication across additional datasets is essential.

A notable strength of this analysis is the use of individual participant-level data from a large clinical trial database. However, there are also limitations. One limitation is that PD discordance was based only on the presence or absence of PD; there was no analysis of whether site and central readers agreed on the date of PD, an important determinant of PFS. In trials without VOP, the investigator may have stopped sending scans for central review when they identified PD, but if another imaging assessment had been sent for central review, BICR may have also identified PD. The potential for different assessors at sites to read the images at different timepoints may increase measured site–central discordance relative to a design in which one assessor performed all reads for a given patient across all timepoints. The lack of a standardized read platform may have contributed to measurement variability between central and local reviewers, although this is representative of real-world practice where multiple PACS vendors and tools are deployed at sites and central core labs. Adjudication rates from the central review are unavailable because only final adjudicated results were provided to the sponsor. This analysis cannot determine whether a given instance of discordance reflects a true error by one reader or a genuine, defensible disagreement between two reasonable interpretations. This is, in part, an inherent feature of the question under study rather than a gap that additional follow-up could fully resolve. Rather than attempting to adjudicate individual cases as correct or incorrect, this analysis characterizes the expected magnitude of discordance itself, providing a benchmark against which observed rates in future trials can be judged. It is also difficult to assess differences in discordance between individual cancer types because the number of trials included for each was small and the magnitude of tumor size changes was not considered. However, cancer types with more non-target disease may show higher rates of PD discordance due to the subjective nature of non-target progression assessment. Moreover, subgroup analyses, especially cross-subgroup comparisons, require careful interpretation considering both observed and unobserved confounding factors. Discordance rates may also differ in solid tumor types not included in this analysis, as well as in hematological malignancies, for which different response criteria are applied. All studies included in the analysis were for pembrolizumab given as monotherapy or in combination with cytotoxic or targeted agents, and while the sources of discordance are generally agnostic to treatment type, it is unknown whether the specific numeric benchmarks we observed would transfer unchanged to trials of cytotoxic or targeted therapies given alone. The one immunotherapy-specific phenomenon that may impact PD discordance rates is pseudoprogression, an uncommon response pattern in which the radiologic tumor burden shows an initial increase before subsequent decrease or stabilization. Although pseudoprogression cannot be excluded as a contributor to the observed discordance, the observation that the lowest PD discordance rate in our dataset was for melanoma, the tumor type with the most extensively documented history of pseudoprogression [18], suggests it was not a primary driver in this analysis. Another example of a possible treatment-specific source of discordance is the cavitation of pulmonary tumors observed with antiangiogenic therapy [22,23]. As a result of cavitation, a lesion responding to antiangiogenic therapy may retain or increase its overall diameter; a size-based response criterion can thus misclassify it, and site and central readers may resolve it differently. Because our dataset does not permit a treatment-specific analysis, we cannot address the impact of cavitation in our analysis, but it does reinforce the caution in directly transferring the numeric benchmarks observed here to studies of treatment classes beyond immunotherapy. Finally, because all studies included in this analysis were conducted in the metastatic setting, results cannot be considered informative for the neoadjuvant or adjuvant settings. In those settings, there would be no discordance caused by differences in choice of followed lesions, but there is more latitude for discordance on whether a radiographic finding represents a new lesion due to the clinical data (e.g., biopsy results) available to the investigator; this risk may be mitigated by including prespecified clinical data in the BICR process.

In summary, this analysis addresses a critical gap in oncology trial methodology by establishing tumor-specific benchmarks for case-level discordance between site and central reviewers of radiographic endpoints based on clinical trials of pembrolizumab. The wide variation across cancer types, from approximately 6% to 23% depending on endpoint and tumor biology, underscores that discordance cannot be meaningfully interpreted without reference to the specific clinical context. These empirical benchmarks offer trial teams an evidence-based framework for distinguishing expected reviewer variability from discordance patterns that may warrant further investigation, supporting more efficient and better-targeted data quality oversight. While the underlying sources of discordance characterized here are not specific to immunotherapy, the reported magnitude of discordance should be applied cautiously to trials of non-immunotherapy agents.

## CRediT authorship contribution statement

**Gregory V. Goldmacher:** Writing – original draft, Methodology, Data curation, Conceptualization. **Cynthia Muller Goldberg:** Writing – original draft, Data curation, Conceptualization. **Leslie Boyle:** Writing – review & editing, Data curation. **Liyi Jia:** Writing – review & editing, Methodology, Formal analysis. **Christine K. Gause:** Writing – review & editing. **Marjorie C. Green:** Writing – review & editing. **Lawrence H. Schwartz:** Writing – original draft.

## Ethics declaration

Written informed consent to take part in the study and to publish the article has been obtained from all participants or their legal representatives. The privacy rights of participants have been observed.

This study was performed in compliance with relevant laws, regulatory frameworks and guidelines where the research took place. This study was approved by the appropriate ethics body for each participating center in each of the 39 clinical trials included in this pooled analysis. (Approval No. NA [no single ethics committee approval number])

The results of this clinical trial and any associated work have been posted in a registry. This clinical trial was registered with number NCT03635567, NCT02460198, NCT02563002, NCT02559687, NCT02564263, NCT03189719, NCT02335411, NCT02370498, NCT02494583, NCT03615326, NCT03675737, NCT02252042, NCT02358031, NCT02702414, NCT02702401, NCT02039674, NCT02142738, NCT02220894, NCT02578680, NCT02775435, NCT03302234, NCT03066778, NCT03631784, NCT01704287, NCT02821000, NCT02752074, NCT03776136, NCT03834493, NCT03834506, NCT04191096, NCT03834519, NCT02853331, NCT02853344, NCT02447003, NCT02555657, NCT02819518, NCT02256436, NCT02335424, NCT02853305 (ClinicalTrials.gov).

## Declaration of generative AI and AI-assisted technologies in the manuscript preparation process

During the preparation of this work, the authors used Claude Opus 4.6 in order to refine the text and clarify the rationale for the analysis. After using this tool, the authors reviewed and edited the content as needed and take full responsibility for the content of the published article.

## Funding

10.13039/100012781Merck Sharp & Dohme 10.13039/100023970LLC, a subsidiary of 10.13039/100004334Merck & Co., Inc., Rahway, NJ, USA, funded all clinical trials included in this pooled analysis. Employees of the sponsor contributed to the design of the individual trials and the analysis and interpretation of their data. Employees of the sponsor are authors of this submission and contributed to the decision to submit this article for publication.

## Declaration of competing interest

The authors declare the following financial interests/personal relationships which may be considered as potential competing interests:Gregory V Goldmacher, Cynthia Muller Goldberg, Leslie Boyle, Liyi Jia, Christine K Gause, and Marjorie C Green are current or former employees of Merck Sharp & Dohme LLC, a subsidiary of Merck & Co., Inc., Rahway, NJ, USA (MSD) and own stock or stock options in Merck & Co., Inc., Rahway, NJ, USA. Lawrence H Schwartz reports funding from Bristol Myers Squibb to support research in imaging biomarkers and providing blinded independent review of imaging studies as part of clinical trials funded by Bristol Myers Squibb, Regeneron, and AbbVie.All authors received medical writing and editorial support provided by MSD.

## Data Availability

Merck Sharp & Dohme LLC, a subsidiary of Merck & Co., Inc., Rahway, NJ, USA (MSD), is committed to providing qualified scientific researchers access to anonymized data and clinical study reports from the company's clinical trials for the purpose of conducting legitimate scientific research. MSD is also obligated to protect the rights and privacy of trial participants and, as such, has a procedure in place for evaluating and fulfilling requests for sharing company clinical trial data with qualified external scientific researchers. The MSD data sharing website (available at: https://externaldatasharing-msd.com/) outlines the process and requirements for submitting a data request. Feasible requests will be reviewed by a committee of MSD subject matter experts to assess the scientific validity of the request and the qualifications of the requestors. In line with data privacy legislation, submitters of approved requests must enter into a standard data-sharing agreement with MSD before data access is granted. Data will be made available for request after product approval in the US and EU or after product development is discontinued. There are circumstances that may prevent MSD from sharing requested data, including country or region-specific regulations. If the request is declined, it will be communicated to the investigator. Access to genetic or exploratory biomarker data requires a detailed statistical analysis plan that is collaboratively developed by the requestor and MSD subject matter experts; after approval of the statistical analysis plan and execution of a data-sharing agreement, MSD will either perform the proposed analyses and share the results with the requestor or will construct biomarker covariates and add them to a file with clinical data that is uploaded to a SAS portal so that the requestor can perform the proposed analyses.
