# Supplementary material for: Setting the standard: empirical benchmarks for case-level site–central discordance in radiographic endpoints in oncology clinical trials
Source: Contemp Clin Trials Commun. 2026 Sep 3;53:101682. doi: 10.1016/j.conctc.2026.101682 (PMC13571531; doi:10.1016/j.conctc.2026.101682)
Supplement: Supplementary file 1 [file mmc1.docx]

**Appendix A.**

**Table A.1.** Summary of clinical trials included in the analysis.

| **Study by Cancer Type** | **ClinicalTrials.gov Number** | **Phase** | **No. Participants Enrolled** | **Treatment Arm(s)** | **Study Design** | **Included VOP** |
| --- | --- | --- | --- | --- | --- | --- |
| **Cervical** |  |  |  |  |  |  |
| KEYNOTE-826 | NCT03635567 | 3 | 617 | Pembrolizumab + chemotherapy vs placebo + chemotherapy | Double blind | Yes |
| **Colorectal** |  |  |  |  |  |  |
| KEYNOTE-164 | NCT02460198 | 2 | 124 | Pembrolizumab | Open label | No |
| KEYNOTE-177 | NCT02563002 | 3 | 307 | Pembrolizumab vs chemotherapy | Open label | Yes |
| **Esophageal** |  |  |  |  |  |  |
| KEYNOTE-180 | NCT02559687 | 2 | 121 | Pembrolizumab | Open label | No |
| KEYNOTE-181 | NCT02564263 | 3 | 628 | Pembrolizumab vs chemotherapy | Open label | Yes |
| KEYNOTE-590 | NCT03189719 | 3 | 749 | Pembrolizumab + chemotherapy vs placebo + chemotherapy | Double blind | Yes |
| **Gastric** |  |  |  |  |  |  |
| KEYNOTE-059 | NCT02335411 | 2 | 318 | Pembrolizumab ± chemotherapy | Open label | No |
| KEYNOTE-061 | NCT02370498 | 3 | 592 | Pembrolizumab vs paclitaxel | Open label | Yes |
| KEYNOTE-062 | NCT02494583 | 3 | 763 | Pembrolizumab ± chemotherapy vs placebo + chemotherapy | Open label (pembrolizumab monotherapy)  Double blind (pembrolizumab + chemotherapy and placebo + chemotherapy) | Yes |
| KEYNOTE-811 | NCT03615326 | 3 | 738 | Pembrolizumab + trastuzumab + chemotherapy vs placebo + trastuzumab + chemotherapy | Double blind | Yes |
| KEYNOTE-859 | NCT03675737 | 3 | 1579 | Pembrolizumab + chemotherapy vs placebo + chemotherapy | Double blind | Yes |
| **Head and neck (squamous)** |  |  |  |  |  |  |
| KEYNOTE-040 | NCT02252042 | 3 | 495 | Pembrolizumab vs standard treatment | Open label | Yes |
| KEYNOTE-048 | NCT02358031 | 3 | 882 | Pembrolizumab vs pembrolizumab + chemotherapy vs cetuximab + chemotherapy | Open label | Yes |
| **Hepatocellular** |  |  |  |  |  |  |
| KEYNOTE-224 | NCT02702414 | 2 | 156 | Pembrolizumab | Open label | No |
| KEYNOTE-240 | NCT02702401 | 3 | 413 | Pembrolizumab vs placebo | Double blind | Yes |
| **Lung** |  |  |  |  |  |  |
| KEYNOTE-021 Cohort G | NCT02039674 | 2 | 123 | Pembrolizumab + chemotherapy vs chemotherapy | Open label | No |
| KEYNOTE-024 | NCT02142738 | 3 | 305 | Pembrolizumab vs chemotherapy | Open label | No |
| KEYNOTE-042 | NCT02220894 | 3 | 1274 | Pembrolizumab vs chemotherapy | Open label | No |
| KEYNOTE-189 | NCT02578680 | 3 | 616 | Pembrolizumab + chemotherapy vs placebo + chemotherapy | Double blind | Yes |
| KEYNOTE-407 | NCT02775435 | 3 | 559 | Pembrolizumab + chemotherapy vs placebo + chemotherapy | Double blind | Yes |
| KEYNOTE-598 | NCT03302234 | 3 | 568 | Pembrolizumab + ipilimumab vs pembrolizumab + placebo | Double blind | Yes |
| KEYNOTE-604 | NCT03066778 | 3 | 453 | Pembrolizumab + EP vs placebo + EP | Double blind | Yes |
| KEYNOTE-799 | NCT03631784 | 2 | 216 | Pembrolizumab + cCRT | Open label | Yes |
| **Melanoma** |  |  |  |  |  |  |
| KEYNOTE-002 | NCT01704287 | 2 | 540 | Pembrolizumab vs chemotherapy | Double blind | No |
| KEYNOTE-151 | NCT02821000 | 1b | 103 | Pembrolizumab | Open label | No |
| KEYNOTE-252 | NCT02752074 | 3 | 706 | Pembrolizumab + epacadostat vs pembrolizumab + placebo | Double blind | No |
| LEAP-004 | NCT03776136 | 2 | 103 | Lenvatinib + pembrolizumab | Open label | Yes |
| **Prostate** |  |  |  |  |  |  |
| KEYNOTE-641 | NCT03834493 | 3 | 1244 | Pembrolizumab + enzalutamide vs placebo + enzalutamide | Double blind | Yes |
| KEYNOTE-921 | NCT03834506 | 3 | 1030 | Pembrolizumab + docetaxel vs placebo + docetaxel | Double blind | Yes |
| KEYNOTE-991 | NCT04191096 | 3 | 1251 | Pembrolizumab + enzalutamide + ADT vs placebo + enzalutamide + ADT | Double blind | Yes |
| KEYLYNK-010 | NCT03834519 | 3 | 793 | Pembrolizumab + olaparib vs abiraterone acetate or enzalutamide | Open label | Yes |
| **Renal cell** |  |  |  |  |  |  |
| KEYNOTE-426 | NCT02853331 | 3 | 861 | Pembrolizumab + axitinib vs sunitinib | Open label | Yes |
| KEYNOTE-427 | NCT02853344 | 2 | 275 | Pembrolizumab | Open label | Yes^a^ |
| **Triple-negative breast** |  |  |  |  |  |  |
| KEYNOTE-086 | NCT02447003 | 2 | 254 | Pembrolizumab | Open label | No |
| KEYNOTE-119 | NCT02555657 | 3 | 622 | Pembrolizumab vs chemotherapy | Open label | Yes |
| KEYNOTE-355 | NCT02819518 | 3 | 882 | Pembrolizumab + chemotherapy vs placebo + chemotherapy | Double blind | Yes |
| **Urothelial** |  |  |  |  |  |  |
| KEYNOTE-045 | NCT02256436 | 3 | 542 | Pembrolizumab vs chemotherapy | Open label | No |
| KEYNOTE-052 | NCT02335424 | 2 | 374 | Pembrolizumab | Open label | No |
| KEYNOTE-361 | NCT02853305 | 3 | 1010 | Pembrolizumab ± chemotherapy vs chemotherapy | Open label | Yes |

^a^Cohort B only.

ADT, androgen deprivation therapy; cCRT, concurrent chemoradiotherapy; EP, etoposide + a platinum; VOP, verification of progression.

**Table A.2.** 2 x 2 table of case-level rates of discordance in progressive disease (PD) assessment between investigators and blinded, independent central radiologists.

|  | | | **Overall** | | **With VOP** | | | | **Trial Design** | | | | **Cancer Type** | | | |
| --- | --- | --- | --- | --- | --- | --- | --- | --- | --- | --- | --- | --- | --- | --- | --- | --- |
|  |  |  |  |  | **No** | | **Yes** | | **Double-Blind** | | **Open-Label** | | **Cervical** | | **Colorectal** | |
|  |  |  | **INV PD** | | **INV PD** | | **INV PD** | | **INV PD** | | **INV PD** | | **INV PD** | | **INV PD** | |
|  |  |  | **Yes** | **No** | **Yes** | **No** | **Yes** | **No** | **Yes** | **No** | **Yes** | **No** | **Yes** | **No** | **Yes** | **No** |
| **BICR PD** | **Yes** | | 11514 | 1256 | 1865 | 173 | 9649 | 1083 | 5961 | 723 | 5553 | 533 | 280 | 16 | 144 | 29 |
|  | **No** | | 2441 | 5697 | 425 | 493 | 2016 | 5204 | 1350 | 3726 | 1091 | 1971 | 77 | 192 | 29 | 167 |
| **Discordance rate, % (95% CI)** | | | 17.7  (17.2-18.2) | | 20.2  (18.8-21.7) | | 17.3  (16.7-17.8) | | 17.6  (16.9-18.3) | | 17.8  (17.0-18.6) | | 16.5  (13.5-19.8) | | 15.7  (12.2-19.8) | |
|  | | | | | | | | | | | | | | | | |
|  | | | **Cancer Type** | | | | | | | | | | | | | |
|  |  |  | **Esophageal** | | **Gastric** | | **Hepatocellular** | | **Head & Neck (squamous)** | | **Lung** | | **Melanoma** | | **Prostate** | |
|  |  |  | **INV PD** | | **INV PD** | | **INV PD** | | **INV PD** | | **INV PD** | | **INV PD** | | **INV PD** | |
|  |  |  | **Yes** | **No** | **Yes** | **No** | **Yes** | **No** | **Yes** | **No** | **Yes** | **No** | **Yes** | **No** | **Yes** | **No** |
| **BICR PD** | | **Yes** | 839 | 54 | 2126 | 182 | 354 | 34 | 801 | 87 | 1976 | 167 | 858 | 86 | 1293 | 404 |
|  |  | **No** | 229 | 197 | 506 | 740 | 90 | 67 | 105 | 178 | 536 | 862 | 73 | 326 | 297 | 2216 |
| **Discordance rate, % (95% CI)** | | | 21.5  (19.3-23.8) | | 19.4 (18.1-20.7) | | 22.8  (19.3-26.5) | | 16.4  (14.3-18.6) | | 19.9  (18.6-21.2) | | 11.8  (10.2-13.7) | | 16.7  (15.5-17.8) | |

|  | | **Cancer Type** | | | | | |  |
| --- | --- | --- | --- | --- | --- | --- | --- | --- |
|  |  | **Renal Cell** | | **Triple-Negative Breast** | | **Urothelial** | |  |
|  |  | **INV PD** | | **INV PD** | | **INV PD** | |  |
|  |  | **Yes** | **No** | **Yes** | **No** | **Yes** | **No** |  |
| **BICR PD** | **Yes** | 531 | 66 | 1207 | 58 | 1105 | 73 |  |
|  | **No** | 119 | 251 | 214 | 165 | 166 | 336 |  |
| **Discordance rate, % (95% CI)** | | 19.1  (16.7-21.8) | | 16.5  (14.8-18.4) | | 14.2  (12.6-16.0) | |  |

BICR, blinded independent central review; CI, confidence interval; INV, investigator; VOP, verification of progression.

**Table A.3.** 2 x 2 table of case-level rates of discordance in objective response (OR) with confirmation assessment between investigator and blinded, independent central radiologists.

|  | | | **Overall** | | **Trial Design** | | | | **Cancer Type** | | | | | | | |
| --- | --- | --- | --- | --- | --- | --- | --- | --- | --- | --- | --- | --- | --- | --- | --- | --- |
|  |  |  |  |  | **Double-Blind** | | **Open-Label** | | **Cervical** | | **Colorectal** | | **Esophageal** | | **Gastric** | |
|  |  |  | **INV OR** | | **INV OR** | | **INV OR** | | **INV OR** | | **INV OR** | | **INV OR** | | **INV OR** | |
|  |  |  | **Yes** | **No** | **Yes** | **No** | **Yes** | **No** | **Yes** | **No** | **Yes** | **No** | **Yes** | **No** | **Yes** | **No** |
| **BICR OR** | **Yes** | | 5967 | 1302 | 3666 | 826 | 2301 | 476 | 300 | 56 | 150 | 12 | 269 | 76 | 1365 | 226 |
|  | **No** | | 1334 | 12917 | 796 | 6942 | 538 | 5975 | 60 | 163 | 35 | 206 | 81 | 902 | 293 | 1911 |
| **Discordance rate, % (95% CI)** | | | 12.2  (11.8-12.7) | | 13.3  (12.7-13.9) | | 10.9  (10.3-11.6) | | 20.0  (16.8-23.5) | | 11.7  (8.7-15.2) | | 11.8  (10.1-13.7) | | 13.7 (12.6-14.8) | |
|  | | | | | | | | | | | | | | | | |
|  | | | **Cancer Type** | | | | | | | | | | | | | |
|  |  |  | **Hepatocellular** | | **Head & Neck (squamous)** | | **Lung** | | **Melanoma** | | **Prostate** | | **Renal Cell** | | **Triple-Negative Breast** | |
|  |  |  | **INV OR** | | **INV OR** | | **INV OR** | | **INV OR** | | **INV OR** | | **INV OR** | | **INV OR** | |
|  |  |  | **Yes** | **No** | **Yes** | **No** | **Yes** | **No** | **Yes** | **No** | **Yes** | **No** | **Yes** | **No** | **Yes** | **No** |
| **BICR OR** | | **Yes** | 57 | 18 | 229 | 74 | 1525 | 313 | 301 | 50 | 441 | 233 | 437 | 78 | 346 | 77 |
|  |  | **No** | 9 | 412 | 74 | 791 | 302 | 1771 | 73 | 838 | 160 | 3382 | 63 | 497 | 75 | 1078 |
| **Discordance rate, % (95% CI)** | | | 5.4  (3.6-7.8) | | 12.7  (10.8-14.7) | | 15.7  (14.6-16.9) | | 9.7  (8.2-11.5) | | 9.3  (8.5-10.2) | | 13.1  (11.2-15.3) | | 9.6  (8.2-11.2) | |

|  | | **Cancer Type** | |  |
| --- | --- | --- | --- | --- |
|  |  | **Urothelial** | |  |
|  |  | **INV OR** | |  |
|  |  | **Yes** | **No** |  |
| **BICR OR** | **Yes** | 547 | 89 |  |
|  | **No** | 109 | 966 |  |
| **Discordance rate, % (95% CI)** | | 11.6  (10.1-13.2) | |  |

BICR, blinded independent central review; CI, confidence interval; INV, investigator.

**Table A.4.** 2 x 2 table of case-level rates of discordance in objective response (OR) without confirmation assessment between investigator and blinded, independent central radiologists.

|  | | | **Overall** | | **Trial Design** | | | | **Cancer Type** | | | | | | | | |
| --- | --- | --- | --- | --- | --- | --- | --- | --- | --- | --- | --- | --- | --- | --- | --- | --- | --- |
|  |  |  |  |  | **Double-Blind** | | **Open-Label** | | **Cervical** | | **Colorectal** | | **Esophageal** | | **Gastric** | | |
|  |  |  | **INV OR** | | **INV OR** | | **INV OR** | | **INV OR** | | **INV OR** | | **INV OR** | | **INV OR** | | |
|  |  |  | **Yes** | **No** | **Yes** | **No** | **Yes** | **No** | **Yes** | **No** | **Yes** | **No** | **Yes** | **No** | **Yes** | | **No** |
| **BICR OR** | **Yes** | | 5877 | 1028 | 3564 | 605 | 2313 | 423 | 354 | 55 | 166 | 17 | 352 | 107 | 1229 | | 180 |
|  | **No** | | 1207 | 7097 | 635 | 2701 | 572 | 4396 | 59 | 111 | 39 | 181 | 109 | 762 | 204 | | 667 |
| **Discordance rate, % (95% CI)** | | | 14.7  (14.1-15.3) | | 16.5  (15.7-17.4) | | 12.9  (12.2-13.7) | | 19.7  (16.5-23.2) | | 13.9  (10.7-17.7) | | 16.2  (14.3-18.3) | | 16.8  (15.3-18.4) | | |
|  | | | | | | | | | | | | | | | | | |
|  | | | **Cancer Type** | | | | | | | | | | | | | | |
|  |  |  | **Hepatocellular** | | **Head & Neck (squamous)** | | **Lung** | | **Melanoma** | | **Renal Cell** | | **Triple-Negative Breast** | | **Urothelial** | | |
|  |  |  | **INV OR** | | **INV OR** | | **INV OR** | | **INV OR** | | **INV OR** | | **INV OR** | | **INV OR** | | |
|  |  |  | **Yes** | **No** | **Yes** | **No** | **Yes** | **No** | **Yes** | **No** | **Yes** | **No** | **Yes** | **No** | **Yes** | **No** | |
| **BICR OR** | | **Yes** | 63 | 19 | 298 | 76 | 1487 | 279 | 340 | 54 | 487 | 75 | 459 | 81 | 642 | 85 | |
|  |  | **No** | 12 | 402 | 99 | 698 | 292 | 1271 | 90 | 774 | 69 | 444 | 103 | 933 | 131 | 854 | |
| **Discordance rate, % (95% CI)** | | | 6.3  (4.3-8.8) | | 14.9  (12.9-17.1) | | 17.2  (15.9-18.5) | | 11.4  (9.7-13.3) | | 13.4  (11.4-15.6) | | 11.7  (10.1-13.4) | | 12.6  (11.1-14.3) | | |

BICR, blinded independent central review; CI, confidence interval; INV, investigator.

**Table A.5.** Directional assessment of discordance between investigator and blinded, independent central radiologists in progressive disease (PD) and objective response (OR).

|  | **PD** | | | **OR With Confirmation** | | |
| --- | --- | --- | --- | --- | --- | --- |
|  | **Total No. Discordant Cases** | **INV-Positive/ BICR-Negative** | **BICR-Positive/ INV-Negative** | **Total Discordant Cases** | **INV-Positive/ BICR-Negative** | **BICR-Positive/ INV-Negative** |
| **Overall** | 3697 | 2441 (66.0%) | 1256 (34.0%) | 2636 | 1334 (50.6%) | 1302 (49.4%) |
| **With VOP** |  |  |  |  |  |  |
| **No** | 598 | 425 (71.1%) | 173 (28.9%) | NA | NA | NA |
| **Yes** | 3099 | 2016 (65.1%) | 1083 (34.9%) | NA | NA | NA |
| **Study design** |  |  |  |  |  |  |
| **Double-blind** | 2073 | 1350 (65.1%) | 723 (34.9%) | 1622 | 796 (49.1%) | 826 (50.9%) |
| **Open-label** | 1624 | 1091 (67.2%) | 533 (32.8%) | 1014 | 538 (53.1%) | 476 (46.9%) |
| **Cancer type** |  |  |  |  |  |  |
| **Cervical** | 93 | 77 (82.8%) | 16 (17.2%) | 116 | 60 (51.7%) | 56 (48.3%) |
| **Colorectal** | 58 | 29 (50.0%) | 29 (50.0%) | 47 | 35 (74.5%) | 12 (25.5%) |
| **Esophageal** | 283 | 229 (80.9%) | 54 (19.1%) | 157 | 81 (51.6%) | 76 (48.4%) |
| **Gastric** | 688 | 506 (73.5%) | 182 (26.5%) | 519 | 293 (56.5%) | 226 (43.5%) |
| **Hepatocellular** | 124 | 90 (72.6%) | 34 (27.4%) | 27 | 9 (33.3%) | 18 (66.7%) |
| **Head & neck (squamous)** | 192 | 105 (54.7%) | 87 (45.3%) | 148 | 74 (50.0%) | 74 (50.0%) |
| **Lung** | 703 | 536 (76.2%) | 167 (23.8%) | 615 | 302 (49.1%) | 313 (50.9%) |
| **Melanoma** | 159 | 73 (45.9%) | 86 (54.1%) | 123 | 73 (59.3%) | 50 (40.7%) |
| **Prostate** | 701 | 297 (42.4%) | 404 (57.6%) | 393 | 160 (40.7%) | 233 (59.3%) |
| **Renal cell** | 185 | 119 (64.3%) | 66 (35.7%) | 141 | 63 (44.7%) | 78 (55.3%) |
| **Triple-negative breast** | 272 | 214 (78.7%) | 58 (21.3%) | 152 | 75 (49.3%) | 77 (50.7%) |
| **Urothelial** | 239 | 166 (69.5%) | 73 (30.5%) | 198 | 109 (55.1%) | 89 (44.9%) |

Percentages are calculated based on the number of discordant cases, not the percentage of all cases. Analysis is unadjusted and descriptive only.

BICR, blinded independent central review; INV, investigator; NA, not applicable; VOP, verification of progression.
